# Bis[6-(cyclo­pentyl­imino­meth­yl)-2-meth­oxyphenolato]nickel(II). Corrigendum

**DOI:** 10.1107/S2056989017006326

**Published:** 2017-05-31

**Authors:** Xiao-Fan Zhao

**Affiliations:** a College of Chemistry & Chemical Engineering, Shaoxing University, Shaoxing 312000, People’s Republic of China

## Abstract

In the paper by Zhao [*Acta Cryst.* (2007), E**63**, m704–m705], the metal atom was reported incorrectly.

## Abstract 2

Erratum to *Acta Cryst.* (2007), E**63**, m704–m705.

The author of Zhao (2007) ▸ has indicated that the metal atom in the reported complex was assigned incorrectly. The paper is therefore withdrawn from the published literature.
